# New analytical methodology for analysing S(IV) species at low pH solutions by one stage titration method (bichromatometry) with a clear colour change. Could potentially replace the state-of-art-method iodometry at low pH analysis due higher accuracy

**DOI:** 10.1371/journal.pone.0188227

**Published:** 2017-11-16

**Authors:** Annukka Santasalo-Aarnio, Istvan Galfi, Jorma Virtanen, Michael M. Gasik

**Affiliations:** Department of Chemical and Metallurgical Engineering, School of Chemical Engineering, Aalto University, Aalto, Finland; RMIT University, School of Science, AUSTRALIA

## Abstract

A new, faster and more reliable analytical methodology for S(IV) species analysis at low pH solutions by bichromatometry is proposed. For decades the state of the art methodology has been iodometry that is still well justified method for neutral solutions, thus at low pH media possess various side reactions increasing inaccuracy. In contrast, the new methodology has no side reactions at low pH media, requires only one titration step and provides a clear color change if S(IV) species are present in the solution. The method is validated using model solutions with known concentrations and applied to analyses of gaseous SO_2_ from purged solution in low pH media samples. The results indicate that bichromatometry can accurately analyze SO_2_ from liquid samples having pH even below 0 relevant to metallurgical industrial processes.

## Introduction

Sulfur dioxide (SO_2_) is a compound present in various processes in industry, particularly in metallurgical processes with sulfuric minerals. At these industries, the detailed detection of SO_2_ from liquid media is vital for process control and for creating thermodynamical equilibrium data for SO_2_ –H_2_O –H_2_SO_4_ systems. In addition, SO_2_ has been widely used as a preservative agent at food industry, where operation is at milder pH conditions than in process industry. Traditionally, S(IV) species has been analyzed from liquid media with a 2-step method by iodometry [1] where S(IV) species are oxidized by iodine with Bunsen reaction:

$$S{\text{O}}_{\text{3}}^{\text{2-}}{\text{+I}}_{\text{2}}{\text{+H}}_{\text{2}}\text{O}↔{\text{SO}}_{\text{4}}^{\text{2-}}{\text{+2I}}^{\text{-}}{\text{+2H}}^{\text{+}}$$ (1)

Excess iodine is added to ensure the full oxidation of S(IV) species that is titrated in a second step with thiosulfate solution

$$\text{2}\;{\text{S}}_{\text{2}}{\text{O}}_{\text{3}}^{\text{2}-}+{\text{I}}_{\text{2}}\to {\text{S}}_{\text{4}}{\text{O}}_{\text{6}}^{\text{2-}}+{\text{2I}}^{\text{-}}$$ (2)

For accurate analysis, the amount of added I_2_ should be substantial to ensure that Eq (1) shifts to the right. However, the molar mass of Iodine (M(I_2_) = 254 g mol^-1^) is large and therefore to oxidize each mol of SO_2_ more than 254 g of iodine is needed leading to a large consumption of reactants that is both expensive and increases need for waste handling.

The iodometric method has been widely used for analyzing S(IV) species from solution samples to determine SO_2_ solubility in water [2] and to sulfuric acid [3]: in these studies the inaccuracy of the iodometry has been noted and the deviation of maximum 3% was reported [2]. Thus, improvements have been suggested to overcome the appreciable errors of iodometry: one suggestion is to add acetaldehyde to the solution that would form α-hydroxyl sulfonic acid (that can be analyzed by potentiometric titration with NaOH [4]), the standard deviation of the results lowered to 0.44%. However, this improvement is well suited for neutral pH but not applicable for low pH solutions due to high initial proton concentration. For the SO_2_ solubility studies on aqueous sulfuric acid other methods than titration have been proposed: from gas stream SO_2_ can be detected by one-canal UV-analyzer [3], spectrophotometer [5], purging though a known concentration of H_2_O_2_ solution [6] or scrubbing through sodium tetrachloromercute (II) and further analyzed with additional steps [7]. In addition, from the lean acid solutions use of enzymes [8,9] and electrochemical methods with screen-printed electrodes [10,11] or with nanoparticles [12] have been reported. Nevertheless, these methods are difficult or impossible to apply solutions at pH below 1 and many of them involve various experimental steps or expensive equipment. These studies highlight a need for simple, reliable and one-step liquid sample analysis method for SO_2_ detection at different pH solutions.

It has already recognized in 1928 that the analysis of iodine in acidic solutions is inaccurate [13]. As low pH values drive the reaction (1) to the left, all S(IV) species are not oxidized and subsequently some I_2_ is not consumed. For this reason the amount of thiosulphate falsely increases in reaction (2), indicating a decrease in the amount of analyzed S(IV) species. If pH of the analyzed solution varies, the SO_2_ detection becomes as a function of solution pH making the result not only false but also unpredicted and scattered. In addition, the excess presence of protons decomposes thiosulphate in parallel, increasing the error of the titration results at low pH:

$$3S_{2}O_{3}^{2-}+2H^{+}\to 4S+2SO_{4}^{2-}+H_{2}O$$ (3)

For all these reasons, analyzing SO_2_ at low pH media with iodometry is slow, unreliable and non-repeatable and therefore, a new analysis method of SO_2_ at low pH media has been sought. Various different oxidants were studied, however, a method with only one titration step, a clear change of colors and with the low consumption of reactants was aimed. Ammonium bicromate showed the most promising results in reaction where bichromate ion oxidizes S(IV) species to S(VI) and the titration can be monitored by changes in the redox potential.

$${\text{Cr}}_{\text{2}}{\text{O}}_{\text{7}}^{\text{2-}}{\text{+3SO}}_{\text{2}}\;\text{(aq.)}{\text{+2H}}^{\text{+}}\to {\text{2Cr}}^{\text{3+}}{\text{+3SO}}_{\text{4}}^{\text{2-}}{\text{+H}}_{\text{2}}\text{O}$$ (4)

The bichromate titration reaction (4) is practically irreversible and therefore the concentrations of the different species have no effect on reaction balance, on the contrary to the reversible Bunsen reaction (1) where the protons are at product side and drive to reaction towards reactants. For this reason, the bichromatometry titration is well suited for low pH media analyses where the high concentration of protons drives the reactions towards wanted direction.

One particular application that needs accurate SO_2_ detection from low pH solutions is a special case of hydrogen production by SO_2_ depolarized electrolyzer (SDE) [14–18]. In general, water electrolyzer splits water with electricity to produce hydrogen and oxygen gases [19], thus in SDE, SO_2_ is added to the anode electrolyte to change the anode reaction where protons and electrons are formed [14]:

$${\text{SO}}_{\text{2}}\;\text{(aq.)}{\text{+2H}}_{\text{2}}\text{O}\to {\text{H}}_{\text{2}}{\text{SO}}_{\text{4}}\text{+2}\;{\text{H}}^{\text{+}}\text{+2}\;{\text{e}}^{\text{-}}$$ (5)

The electrons attracted by the positive anode and move through external load to the cathode while the positively charged protons move across a polymer electrolyte membrane also to the cathode where they meet the electrons to produce hydrogen gas

$$2{\text{H}}^{\text{+}}{\text{+2e}}^{\text{-}}\;\to \;{\text{H}}_{\text{2}}$$ (6)

With excess SO_2_ in the anolyte stream, the non-charged small SO_2_ molecule has a tendency to carry-over thought the polymer electrolyte membrane used as a separator between anolyte and catholyte streams [20]. If any SO_2_ is present at the cathode, it will reduce to form H_2_S or elemental sulfur. Both of these parasitic products are harmful for the SDE operation and for that reason the accurate detection of SO_2_ from both electrolytes is vital for process optimization. In this particular application, pH of the electrolytes from which the SO_2_ is analyzed is very low (often < 0) and during SDE operation more acid is produced (reaction 5) further decreasing the pH. In addition, in anolyte SO_2_ is very concentrated (200–400 mM) and in catholyte very diluted (0–40 mM). Thus, in an optimal case single titration solution would be preferred. During operation, these samples are analyzed in parallel and there is no time for changing the titration solution concentration.

In this paper, a new titration method for S(IV) species analysis is introduced and validated with model samples. This paper aims at accurate low and high concentrated S(IV) analysis from acidic solutions relevant to metallurgical sulfuric acid and SDE processes and the new method is compared with currently used state-of-the-art method, iodometry, evaluating the strengths and weaknesses of each method. In addition, this work demonstrated the applicability of these both methods for the analysis of dissolved SO_2_ at different low pH media.

## Materials and methods

### Chemical and materials

Chemicals used for the experiments were: Na_2_SO_3_ (p.a. Merck), (NH4)2Cr2O7 (p.a. Sigma-Aldrich), H_2_SO_4_ (98%, Sigma-Aldrich), readymade solutions: 0.5 M I_2_ (Merck), 0.1 M Na_2_S_2_O_3_ (Merck) and 0.5 M NaOH (p.a. Sigma-Aldrich). All solutions were prepared by diluting with Milipore-Q water (< 0.5 μS cm–2) and purged with N_2_ (99.99% AGA-Linde, Finland) for oxygen removal. Experiments with dissolved SO_2_ in acid solutions two different SO2 gas strengths were used to obtain high and low concentrated solutions: Strong gas was (99.98% SO_2_, AGA-Linde, Finland) and the diluted gas (1% SO_2_, 99% N_2_, AGA-Linde, Finland).

### SO_2_ analysis from Na_2_SO_3_ solutions

For model solutions to obtain accurate amount of S(IV) species, Na_2_SO_3_ was dissolved to MQ water and before the experiment added to the low pH solution. MQ water was first purged with N_2_ for 45 min to ensure the removal of oxygen that could react with Na_2_SO_3_ decreasing the amount of S(IV) species in the solution. The samples for analysis were taken within 3 h of the solution preparation and in each titration, fresh solutions were used. All the samples were analyzed for S(IV) species by an automatic titrator Titroline 5000 (from Titrilab) with dosing accuracy systematic error 0.15% and random error 0.05%. In this system a potentiometric electrode (PT 6880, from SCHOTT instruments) was used as a redox electrode to determine the mV end-point. The limit of detection (LoD) for both analysis compared is dependent on the analysis equipment accuracy.

The procedure for Iodometry was to add 110% of the needed iodine into 5 mL of sample and beaker filled with water to obtain the total volume of 30 mL. The sample was stirred with a magnetic stirrer and titrated with 0.1 M thiosulfate solution. Whereas for the Bichromatometry two different procedures were applied: 1) Direct titration by adding 5 mL sample to a beaker with 10 mL of water and this solution was titrated dropwise with fresh 0.05 M bichromate solution. 2) Indirect titration by adding first 70% of the calculated amount of bichromate to a beaker, 5 mL sample was added on top of this reactant followed by dropwise titration with 0.05 M bichromate solution.

### SO_2_ analysis from SO_2_ purged solutions

In the industrial context, SO_2_ produced is in a gas from and to eliminate emission into the air SO_2_ is purged to a solution and further treated. Due to the different nature of analysis S(IV) species from purged SO_2_ solutions in comparison to analysis of S(IV) from dissolved salt solutions, the suitability of the methods to accurate analyze purged SO_2_ solutions was studied. For these experiments, SO_2_ was purged into different concentrated sulfurc acid solutions and saturated levels were used to guarantee the repeatability of the experiments. Firstly, the acid solution was prepared by diluting concentrated H_2_SO_4_ solution with MQ water and then purging for 45 min with N_2_ to remove oxygen. Secondly, the prepared solution was purged with SO_2_ gas (6 l h^-1^ rate) at least for 90 min before sampling to obtain the saturation level and purging continued through the experiment. Followed by analysis with both bichromatometry and iodometry from the same solution with a following procedure: the first analysis of 10 parallel samples with bichromatometry and the secondly analysis of 10 parallel samples with iodometry. At the end, additional three titrations with bichromatometry were performed to confirm that there were no changes in the concentration during the experiment.

## Results and discussion

### Iodometry

For iodometric analytical methodology, a large concern is that the method involves various reactants and steps where possible side reactions mentioned in the introduction can occur. In addition, when part of the iodine has dissolved in water, it can react with non-dissolved iodine and form a polyiodide ion

$${\text{I}}_{2}{\text{+I}}^{\text{-}}\;↔\;{\text{I}}_{\text{3}}^{\text{-}}$$ (7)

Indicating that the titration reagent (0.5 M I_2_ solution) is partially dissociated having an effect on the Bunsen reaction (1) where I^-^ ions are also formed. If this occurs, the consumption of I^-^ ions shifts the Bunsen reaction back to reactants, resulting in errors for all iodine titrations regardless of pH. At low pH, the situation becomes more difficult as excess protons also shift the reaction (1) to the left. For comparison, before testing the method at low pH, iodine titration for different Na_2_SO_3_ concentration in pure MQ water solutions was performed and the results are presented in Table 1.

**Table 1:** Iodometry results for different Na_2_SO_3_ concentration in water, at pH 5–7, the average value and standard deviation.

Sample	50 mM	100 mM	500 mM	1000 mM
1	49.6	97.8	483	978
2	49.6	98.0	470	983
3	49.2	97.6	472	981
4	49.8	98.3	479	982
5	50.0	97.4	473	977
Average	49.6	97.8	475	980
St. Deviation	0.3	0.3	5	3

As can be seen from Table 1 the iodometry provides constant results at water solutions with S(IV) species with standard deviation below 1%. These results imply that when S(IV) species come from Na_2_SO_3_ salt the deviation is smaller than what reported with gaseous SO_2_ in water solutions [2]. Moreover, Table 1 also shows that all the values obtained are below the expected concentration due to the loss of the gaseous product during sampling and stirring. This study confirms that for pH 5–7 solutions iodometry provides acceptably and reasonable values. At low pH media, the effect of iodine autocatalysis (7) and the presence of protons can cause error for the iodometry analysis. To study this effect, two different acid concentrations (1 and 5 M H_2_SO_4_) without any S(IV) species were prepared and analyzed with iodometry (Table 2).

**Table 2:** Iodometry analysis of S(IV) amount at samples on pure acid solution (no S(IV) species present) with different acid concentrations.

	Iodometry / mM	
c (H_2_SO_4_) / M	sample 1	sample 2
1	1.3	2.9
5	1	2.6

As can be seen from Table 2 in pure acid solution a false result that S(IV) species would be present was obtained. It would seem that the results are scattered and are regardless of the acid concentration and would further indicate that iodometry is inaccurate for trace amount titration at low pH solutions that is an important case for the SDE catholyte stream analysis. In samples with S(IV) species are present these same side reactions take place and cause systematic error. In addition, there was another aspect of iodometry noted during titration with 5 M H_2_SO_4_ solution: a large quantity of solid, visible sulfur was formed by the side reaction between thiosulfate with protons in reaction (3) or between thiosulfate with iodine:

$${\text{I}}_{\text{2}}{\text{+4S}}_{\text{2}}{\text{O}}_{\text{3}}^{\text{2-}}↔{\text{3SO}}_{\text{4}}^{\text{2-}}{\text{+5S}}^{\text{0}}{\text{+2I}}^{-}$$ (8)

Large amount of solid sulfur in the sample solution resulted as more difficult and inaccurate analysis. For all these reasons mentioned the iodometry is not suitable for an accurate titration of S(IV) species at low pH media.

### Bichromatometry

**Fig 1:**
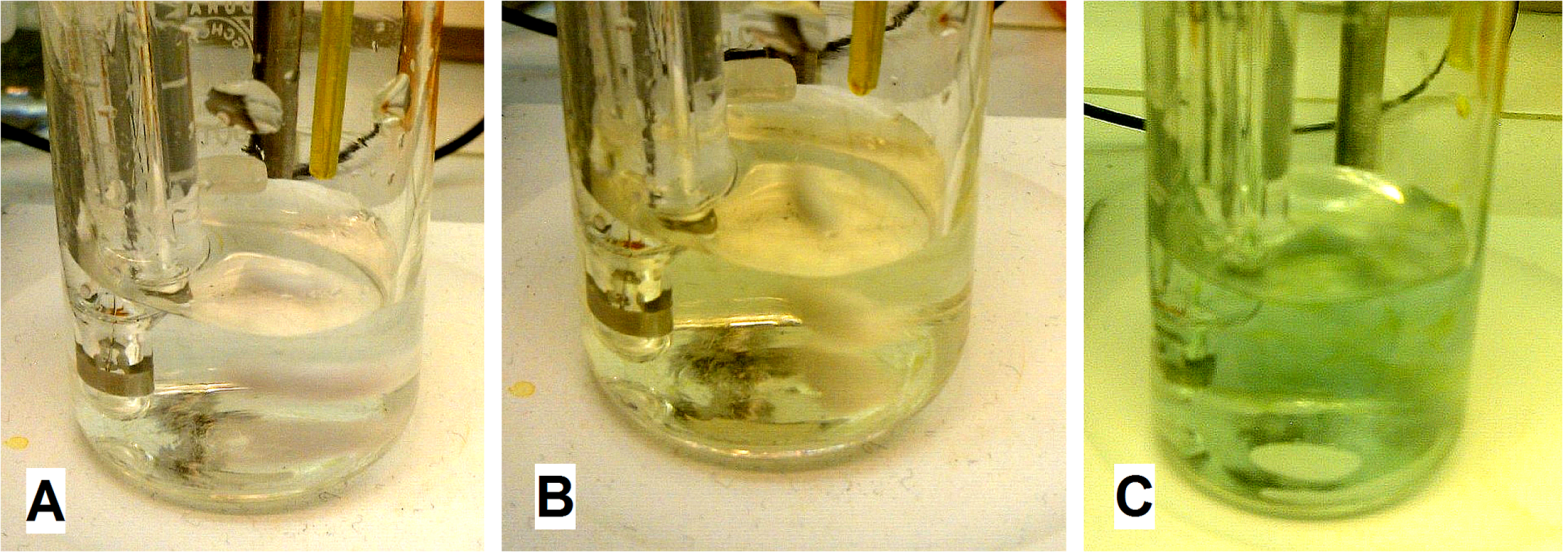
(A) A reference sample of acid solution before bichromate addition. Bichromatometry samples with no S(IV) species (B) and in the presence of S(IV) species (C) after the addition of the first drops of bichromate reagent.

The bichromate titration method was developed with the aim having only one reagent and titration step, notable color change and no side reactions at low pH. The most interesting feature in bichromatometry is a clear color change that indicates to the user if there is any detectable SO_2_ in the solution. With an addition of the first reactant drop in bichromate titration, the clear solution of pure sulfuric acid (Fig 1A) turns to yellow (Fig 1B) indicating that there are no sulfites in the solutions. In the presence of S(IV) species after the first drop the clear solutions turns green: light green if there is trace amount of SO_2_ (Fig 1C) and dark green for high SO_2_ concentration. For the user, the instant color change provides a fast indication if the sample should be analyzed in more detail.

As was seen in Table 2, in pure acid samples iodometry falsely showed traces of S(IV) species when bichromatometry was applied for these same samples the solution turned yellow after a few drops of reactant (Fig 1B) and the titration automatically stopped correctly implying that there were no S(IV) species in the solution. In iodometry, the first reagent is added directly to the sample that oxidizes instantly the S(IV) species but the concentration of the sample should be well known to add correct amount of iodine. Bichromatometry can use the direct dropwise reagent addition that provides possibility to also analyze unknown samples. Nevertheless, the direct method prolongs the analysis time that can lead to larger experimental error due to the evaporation of SO_2_. Uniquely, for bichromatometry it is also possible to use the indirect method where part of the reactant is added directly and then dropwise titrate the remaining species improving the reliability of the analysis without accurate knowledge of the sample initial concentration.

### Validation of the method with sulfite

**Fig 2:**
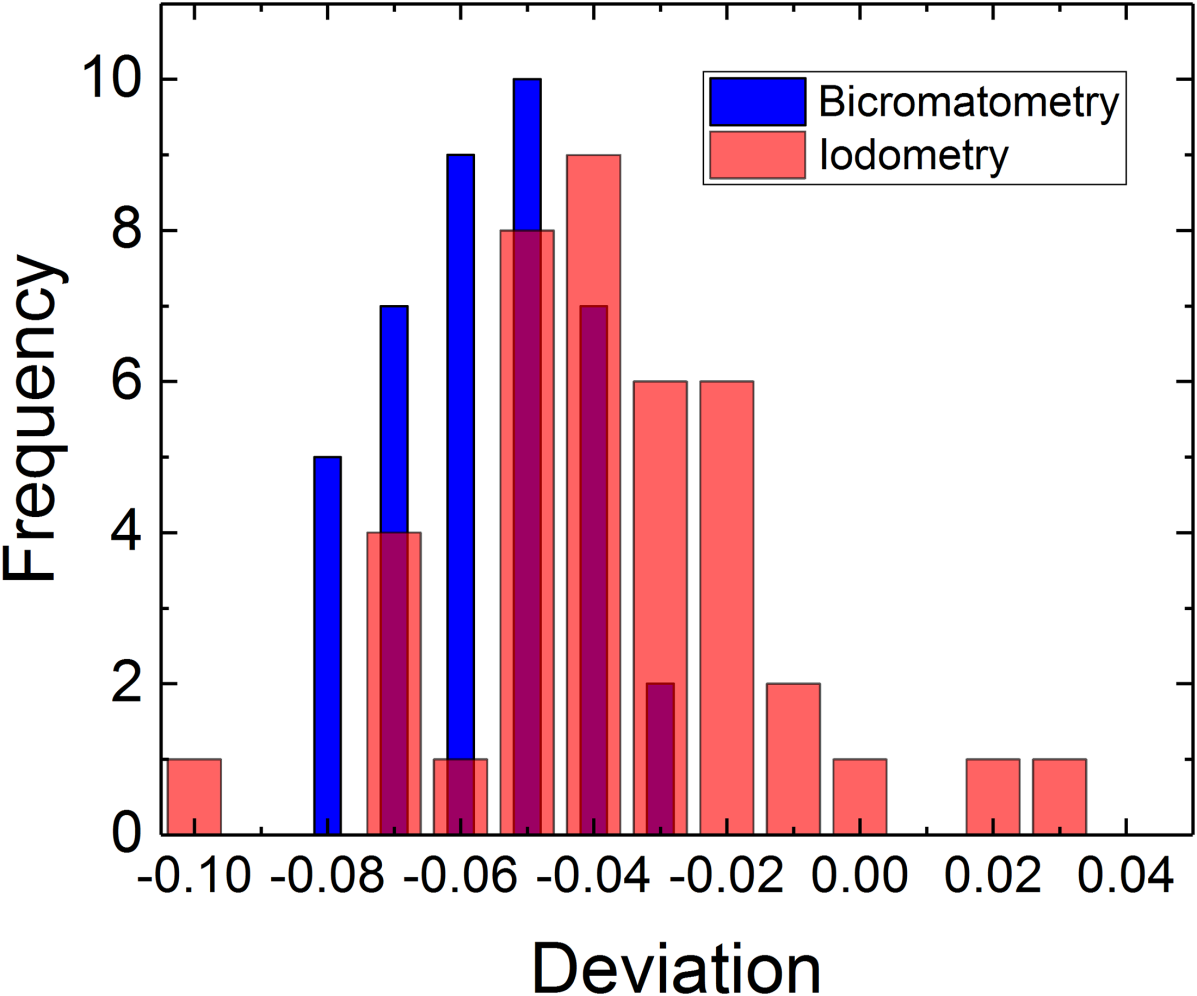
The deviation of the titration results from theoretically expected values with the two different titrations methods for all tested Na_2_SO_3_ concentrations at 1M H_2_SO_4_ solution with pH = -0.2.

The validation of the method was performed with the model solutions of exact S(IV) concentration prepared with Na_2_SO_3_ salt. To observe the concentration dependence on the titration results, four different S(IV) concentrations were studied (50, 100, 150 and 200 mM) in 1 M H_2_SO_4_ solution with pH = -0.2. With both methods, 10 samples were analyzed and the bichormate titrations have been performed with the indirect titration method. Table 3 presents the analysis values and Fig 2 visualizes their deviation from prepared concentrations.

**Table 3:** The titration results for both titration methods at various S(IV) species concentration at 1 M H_2_SO_4_ solution, pH = -0.2.

	50 mM	S(IV)	concentration	
		100 mM	150 mM	200 mM
Iodometry	49.3	95.7	142.9	194.3
	51.0	94.7	144.1	188.1
	51.4	94.9	143.1	196.8
	47.4	94.3	145.8	193.5
	48.7	97.5	143.5	195.8
	46.6	96.9	143.5	193.1
	43.8	92.1	143.7	199.6
	48.3	92.8	139.2	191.9
	47.3	97.2	141.3	192.7
	47.9	94.6	139.4	191.6
Bichromatometry	46.5	93.6	144.9	189.0
	46.4	94.8	144.9	189.3
	45.8	93.0	144.0	188.1
	45.6	93.9	143.4	189.6
	46.5	94.2	142.8	189.9
	46.5	92.4	143.7	189.9
	46.7	92.7	142.8	187.5
	45.8	93.6	142.8	187.5
	45.8	93.3	141.6	189.3
	45.9	93.3	144.0	187.8

From Fig 2 it is evident that almost all results obtained with both methods are below the expected concentration value due to the rapid SO_2_ oxidation and possible losses of the gaseous compounds with stirring. The majority of the results with bichromatometry have narrow distribution, deviation below 3% each other, whereas with the iodometry titration results deviate max. 7% from each other but even -10% to +3% from the initial concentration. This large deviation of the experimental results confirms that the possible side reactions do not create systematic error but instead scattered data sets making reliable analysis impossible. The results highlight that for iodometry analysis to obtain reliable results high amount of experimental points is required that is time and reactant consuming. With bichromatometry consistent and reliable results can be obtained even at low pH solutions with few analysis points.

For a numerical comparison of the deviations, data of Fig 2 were attempted to fit first with a Gaussian distribution, which assumes normality of the error (PeakFit 4.1 software, SPSS Inc.). However, such fit was not possible due singularity of the correlator matrix, even when applying to semi-discrete data. The simplest yet reasonable fitting function found to be gamma-amplitude statistic fit:

$$y(x)=a_{0}\text{exp}\left(-\frac{x-a_{1}}{a_{2}}\right)\;{\left(\frac{\frac{x-a_{1}}{a_{2}}+a_{3}-1}{a_{3}-1}\right)}^{a_{3}-1}$$ (9)

where x variable is the deviation (Fig 2) and y variable is the number of occurrences (Fig 2), treated as a continuous function. The comparison was aimed to fit these functions for both cases, reaching the same level of correlation, standard error and statistical F-values, the fitted plots for iodometry and bichromatometry (Fig 2) are presented in S1 and S2 Figs (supporting information), respectively. The calculated peak parameters from S1 and S2 Figs are presented in Table 4.

**Table 4:** The peak parameters for both titration method and fitted to S1 and S2 Figs (data from Fig 2).

Peak parameters	Amplitude a_0_	Center a_1_	Width a_2_	Shape a_3_
Iodometry (peak 1)	2.7836	-0.06885	0.003394	7.1265431
Iodometry (peak 2)	8.30989312	-0.04024	0.008886	3.8462647
Iodometry (peak 3)	0.80988475	0.025749	0.002198	9.8161757
Bichromatometry (one peak)	9.67284047	-0.05708	0.001297	167.92035

The results in S1 Fig show that iodometry data of Fig 2 require at least three peaks to reach degree-of-freedom adjusted correlation coefficient r^2^ > 0.98. For bichromatometry, only one single peak fit is required to describe data of Fig 2. Thus the stability of the biochromatometry results is significantly better than from a scattered iodometry titration. Table 4 concludes the obtained peak parameters from the fit in S1 Fig and emphasises the accuracy of the bichromatometry.

### Comparison of the methods

To compare the new method for the state-of-art-method iodometry there are few further issues that need to be taken into consideration. Firstly, especially dissolved SO_2_ in water is not in equilibrium and the concentration can vary by the time. Moreover, S(IV) has a tendency to oxidize by time and for the accurate analysis of the S(IV) species, time is an important variable. As was observed in the Fig 2 with neither method the absolute prepared S(IV) amount was observed due to the fast oxidation and loss of the gaseous component. Thus, for repeatable, accurate analysis fast analysis is absolute advantage. Iodometry includes two separate titration steps, that both require time. In case, the sample is not well known, there might be wrong iodine amount added in the first titration stage that can cause the second titration stage to provide 0 result (if there was no iodine left). This leads to a need to repeat the analysis. In the opposite case, if the iodine addition was overestimated, the titration stage prolongs and the reliability of the results suffer. Even though, there would be the perfect amount of iodine added at the first titration step, this method requires two separate titration steps indicating that the minimum time consumed for this titration is twice the amount for the bichromatometry titration.

Second issue that is good to address is the different cost associated with these methods. With bichromatometry only one chemical is needed whereas in the iodometry two different titration step require two different chemicals. As in the latter case the need for chemicals is not directly dependent to the amount of sample but there is a need to have additional iodine (normally 5–10%) added at the first step. The more additional iodine is added to the first step, the more the second chemical thiosulpfate are needed. To avoid repeating the iodometry titration procedure, even excess additional iodine should be added, further adding the cost. With this analysis, iodometry is not only 2 times but even 2.3 times more expensive than bichromatometry from chemicals only.

Moreover, if the sample interval is frequent, in the case of two-step iodometry there might be a need to hire another staff member to perform the second titration. Two-step titration processes are very labor intensive and the possibility of an error increases significantly. As was also stated in S2 Fig and Table 4 from the statistical analysis calculated from the Fig 2 bichromatometry data has better accuracy and therefore, there is a need for a few samples. In case where there is a need to repeat a sample, the same staff has time to repeat the same sample before the composition has changed due to S(IV) oxidation or gas loss. Overall, it is notable that in response of accuracy, analysis time or cost the bichromatometry provides clear improvement for the old state-of-art-method iodometry.

### S(IV) analysis in SO_2_ solutions

**Fig 3:**
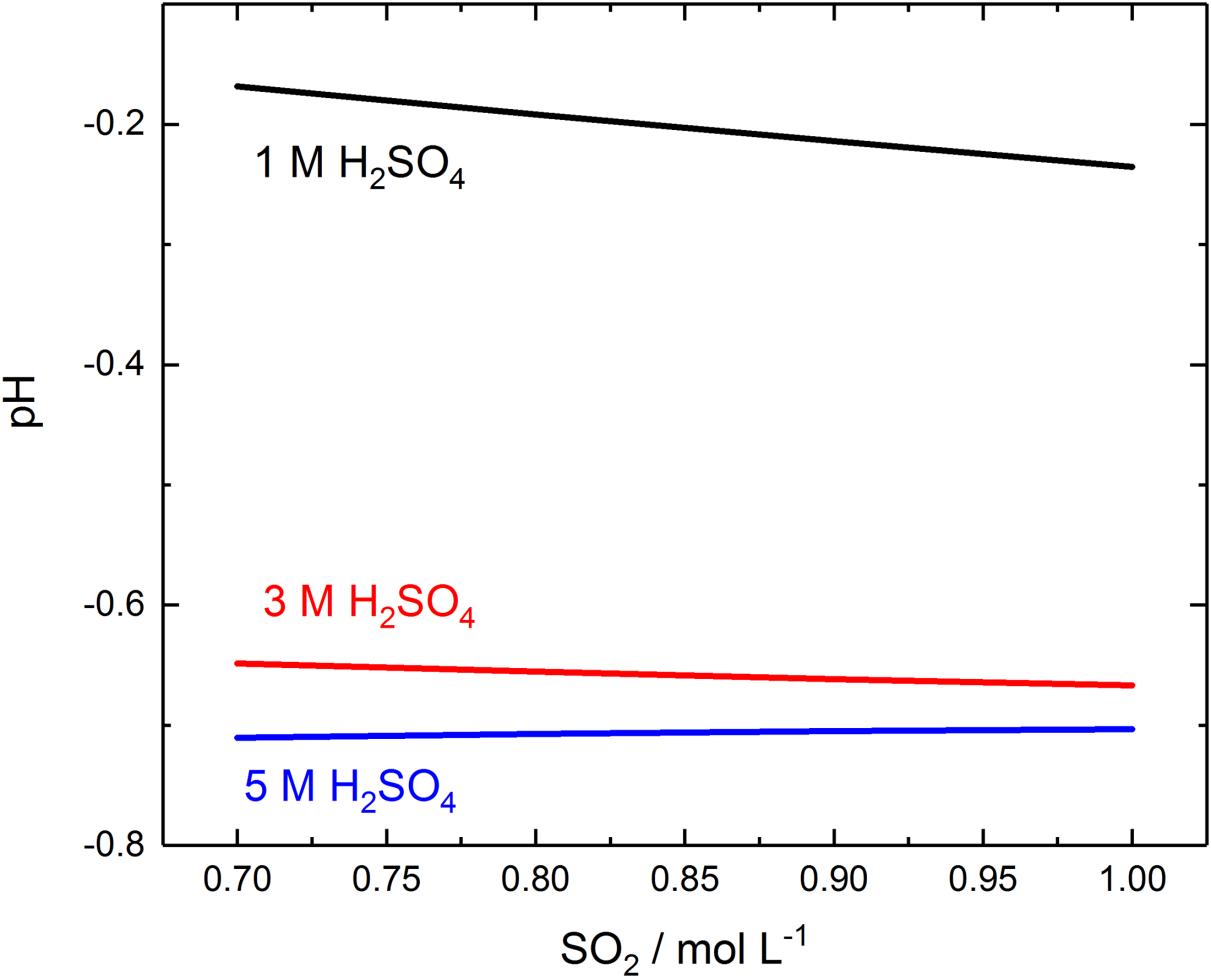
The effect of the dissolved SO_2_ amount to solution pH with different initial acid concentrations.

For industrial systems like SO_2_ –H_2_O –H_2_SO_4_ and SDE, the analyzed samples have typically pH lower than one and have been created by purging gaseous SO_2_ to the liquid phase. Therefore, it was vital to test the applicability of these analysis methods also for systems with dissolved SO_2_ and two different S(IV) levels were obtained purging with 1) diluted SO_2_ gas (1%) in N_2_ or 2) stronger nearly 100% SO_2_ gas. It is known that the efficient SO_2_ dissolution depends on solution pH: the solubility of SO_2_ decreases in lean H_2_SO_4_ solutions in comparison to pure water [6] due to the increase amount of protons that suppress the hydrolysis of dissolved SO_2_ [21]. When acid concentration is further increased, the dissolution improves [22]. Ultimately, the amount of dissolved SO_2_ is a function of solution pH. Nevertheless, as can be seen from calculations results (FactSAGE ver. 6.0 with the Pitzer AQUA database and all stable sulfur compounds included) in Fig 3, increasing the SO_2_ amount in the solution will also have an effect on the solution pH. Particularly, this effect is strong with 1 M H_2_SO_4_ solution where the change of pH is almost 30% when the amount of dissolved SO_2_ changes from 0.7 to 1 M. At stronger acids, this phenomenon is less prone due to higher initial proton concentration.

Dissolved gaseous SO_2_ in sulfuric acid concentrations is present as three major tetravalent sulfur species: SO_2_ (aq.), bisulfite $HS{\text{O}}_{\text{3}}^{\text{-}}$ and sulfite $S{\text{O}}_{\text{3}}^{\text{2-}}$. The current literature describes that the relative concentrations of these species are dependent on the solution pH [23]. Yet, this model is over simplified because as can be seen at Fig 3 also the pH changes with the increase of S(IV) species if all stable S species are included. At 3–5 M H_2_SO_4_ solutions with lower pH -0.6 to -0.7, this effect is less prone with added SO_2_ gas. Nevertheless, pH of any solution is not an independent variable and cannot be changed without the addition of counter ions as the solution electroneutrality must be hold at all times and thus more complex models are needed.

**Fig 4:**
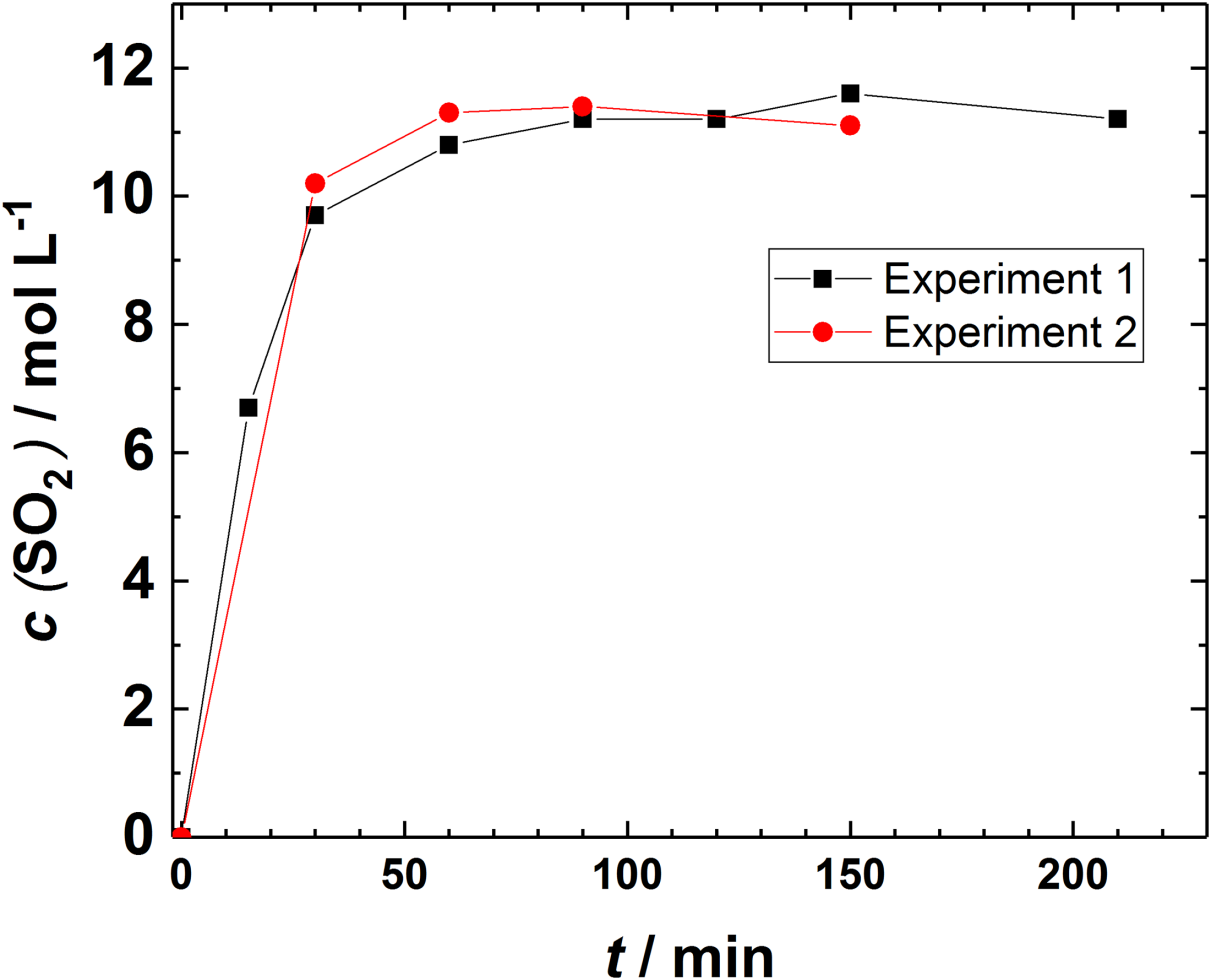
Purging 1% SO_2_ gas to 1 M H_2_SO_4_ solution with pH = -0.2. The points refer to titration values and the lines are added as a guide for the eye.

Prior to experiments a separate study to analyze the SO_2_ saturation level was performed: deoxidized solution was purged with the SO_2_ and samples from the bottle were collected and titrated by bichromatometry to obtain information when the saturation is reached. One of these experiments is presented in Fig 4 where the case of dilute SO_2_ gas (1% SO_2_) was purged to 1 M H_2_SO_4_ solution (experiment 1) and repeated (experiment 2). Fig 4 shows that the saturation level is obtained around 60 min of purging followed by a steady concentration at least for 180 min, thus, to ensure stable results samples were taken after 90 min purging. This confirms that the concentration levels between different experiments are comparable (Fig 4).

**Fig 5:**
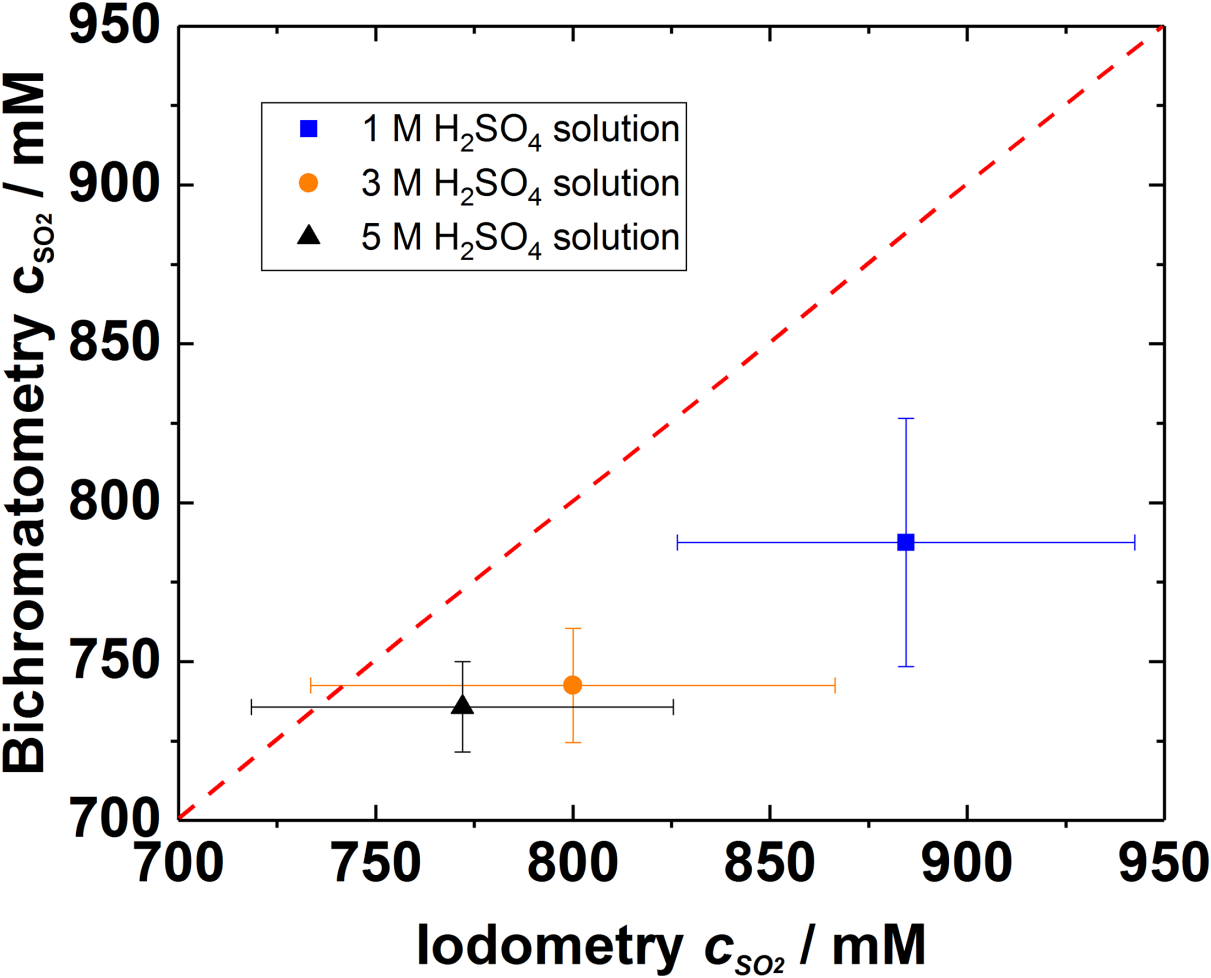
Titration analysis with two different methods for the samples saturated with 100% SO_2_ gas at H_2_SO_4_ solutions with various pH values. The points mark for average of 10 samples and lines indicate the deviation. Different colours indicate the acid concentration.

**Fig 6:**
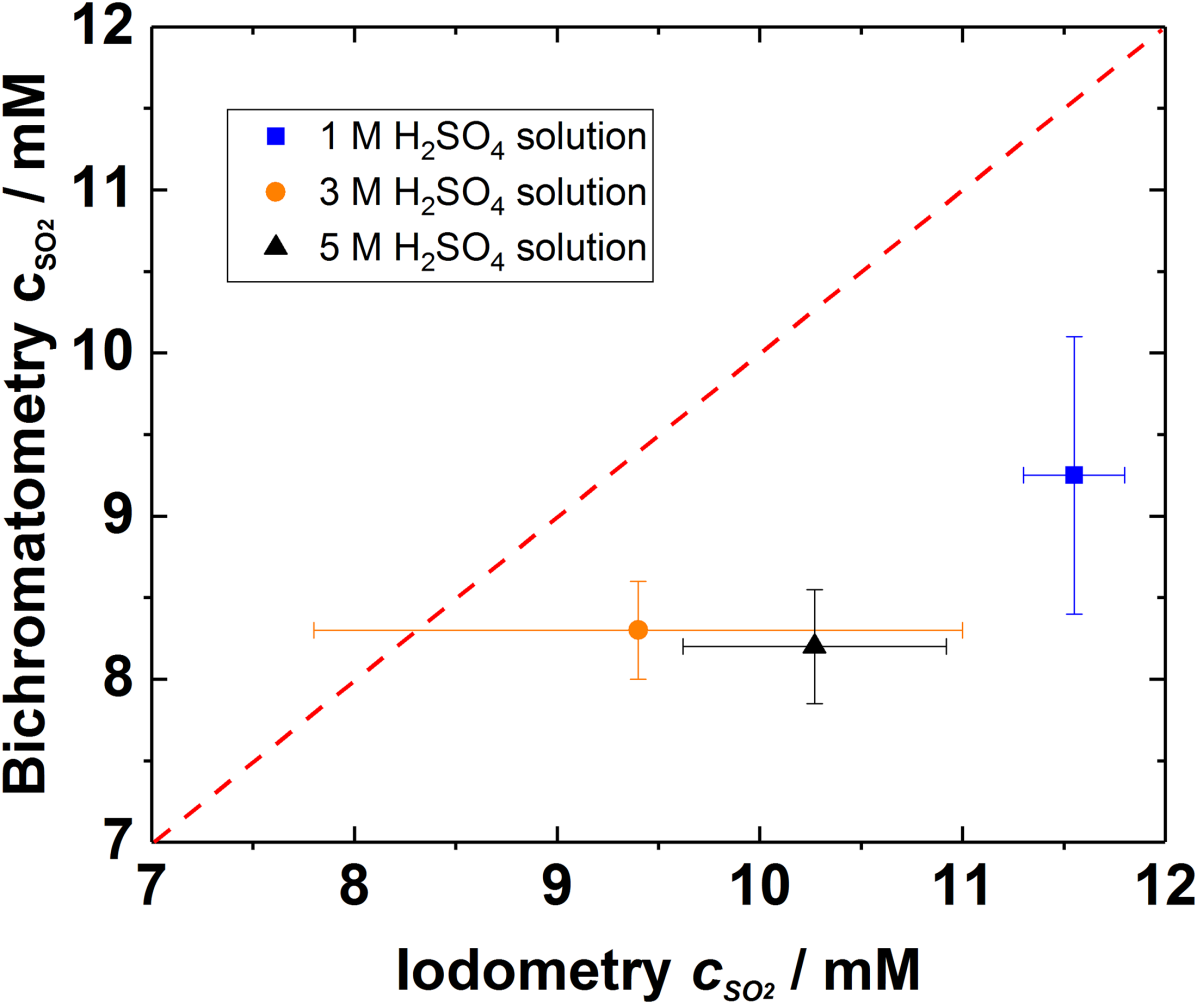
Titration analysis with two different methods for the samples saturated with 1% SO_2_ gas at H_2_SO_4_ solutions with various pH values. The points mark for average of 10 samples and lines indicate the deviation. Different colours indicate the acid concentration.

Fig 5 presents the titration results for the purged strong SO_2_ gas at various acid concentration solutions, this particular case is very relevant in both anolyte stream for SDE and metallurgical processes in sulfuric acid production. Because there is no independent data of the real concentration of the SO_2_ in the purged solutions available, two methods are compared with each other: if both methods provided the same value, the point would be at the dashed slope line (Figs 5 and 6). In Fig 5 it can be clearly seen that highest SO_2_ concentrations are obtained at the highest pH and the amount of dissolved SO_2_ decreases with a decrease of pH [22]. The numerical values obtained with iodometry provides larger values than bichromatometry, eventually due to the side reactions (2), (3), (8), (9) and (10). In addition, the deviation of the titration results is 20–50 mM and 120–150 mM for the bichromatometry and iodometry, respectively (Fig 5) indicating that iodine results deviate even three times more than the results obtained with bichromatometry. In the case of bichromatometry the most consistent results are obtained in the lowest pH solution but with iodometry the deviation in this case is significant. To observe the performance of the titration methods with dilute SO_2_ concentrations (for instance in the case of catholyte stream in SDE), the solutions purged with 1% SO_2_ gas were also examined (Fig 6).

In the case of SDE catholyte stream very diluted SO_2_ gas should be accurately detected in acid solution and this case is presented in Fig 6. The trend in Fig 6 is similar than what observed with concentrated SO_2_ solutions in Fig 5: iodometry overestimates SO_2_ amount in the solutions due to the side reactions of the reactants. In addition, the deviation of the results with iodometry is higher at very concentrated solutions (pH < -0.5). However, at 1M H_2_SO_4_ solution iodometry provides more consistent results than at more concentrated acids. This could indicate that the decomposition of thiosulfate (according to reaction 3) becomes especially severe issue at higher acid concentrations. Whereas the bichromatometry benefits higher proton concentrations and shows more repeatable results at lower pH media.

This method has been applied for the unknown samples of both anolyte and catholyte streams of the SDE [20,24] and indeed the same titration solution can be applied for both cases. As the samples are taken with a short interval (15–30 min), a fast analysis system is needed and bichromatometry can provide that due to only one titration step. The reduced number of operations means not only less error but also less chemicals used and respectively chemical waste produced, which in volume of a large laboratory may have substantial economic impact.

## Conclusion

The methodology developed in this paper aimed at simplified and repeatable analysis method for determine S(IV) species in aqueous sulfuric acid solutions that are common at metallurgical industry processes. An original approach with one-step direct titration method, bichromatometry, is proposed and validated for known S(IV) systems and thereafter tested with industrially representative samples having dissolved gaseous SO_2_ at low pH solutions. The advantages of this new titration method are a visible color change in the presence of S(IV) species and need of only one titration step with one reactant that provides a fast and reliable analysis of unknown samples and low consumption of reactants. The results with model solutions confirm that the deviation of the titration results from the initial concentrations was significantly lower with bichromatometry in comparison to the state-of-art-method iodometry. In addition, trace amount SO_2_ can be analyzed with bichromatometry due to the clear color change indicating a need for further processing of the sample. At low pH solutions purged with gaseous SO_2_ bichromatometry provided less deviating titration values in both diluted and strong SO_2_ concentrations particularly at most acidic solutions. Overall, bichromatometry provides a repeatable, fast titration method from liquid samples that offers significant savings on reactants, analysis time and increases the accuracy of the SO_2_ analysis at low pH media.

## Supporting information

S1 Fig Fitting of the models on the iodometry data from Fig 2 from the manuscript. (PDF)

S2 Fig Fitting of the models on the bichromatometry data from Fig 2 from the manuscript. (PDF)
