# Supplementary figures and images for: New analytical methodology for analysing S(IV) species at low pH solutions by one stage titration method (bichromatometry) with a clear colour change. Could potentially replace the state-of-art-method iodometry at low pH analysis due higher accuracy
Source: PLoS One. 2017 Nov 16;12(11):e0188227. doi: 10.1371/journal.pone.0188227 (PMC5689830; doi:10.1371/journal.pone.0188227)

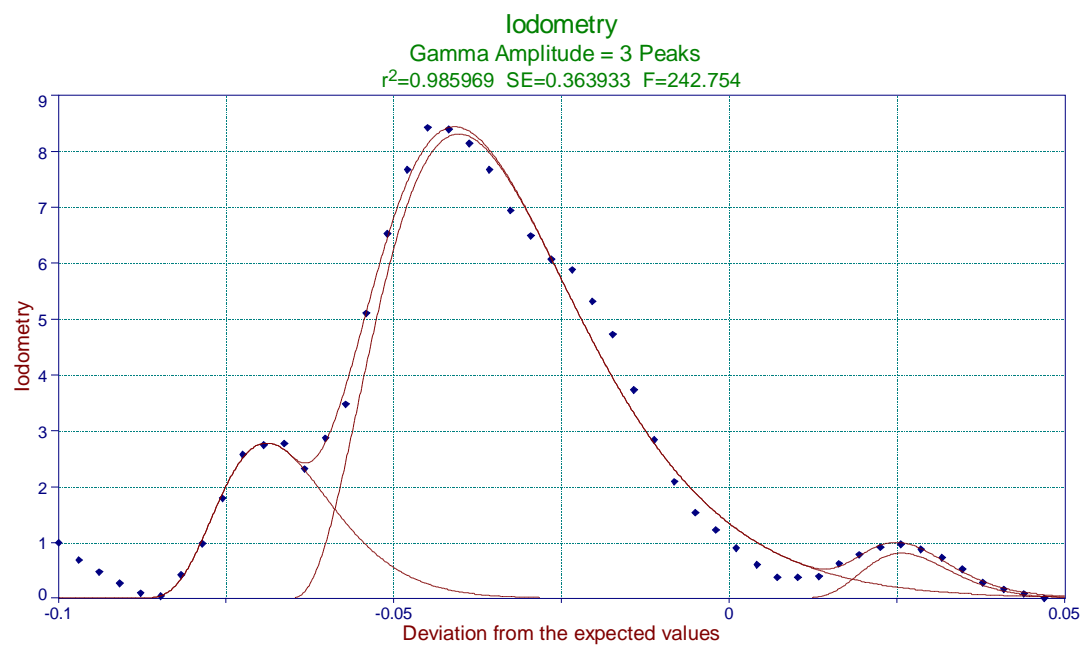

Supplement: S1 Fig — (PDF) [file pone.0188227.s001.pdf]

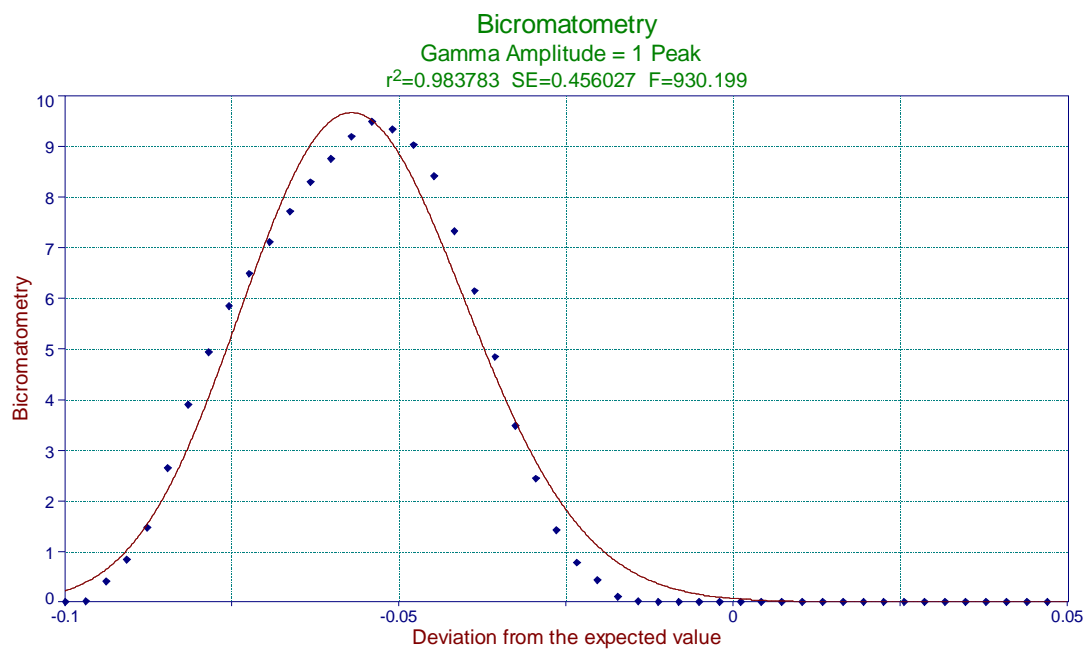

Supplement: S2 Fig — (PDF) [file pone.0188227.s002.pdf]
